# Effectiveness of corticosteroid in the treatment of dengue – A systemic review

**DOI:** 10.1016/j.heliyon.2018.e00816

**Published:** 2018-09-22

**Authors:** S.M. Rathnasiri Bandara, H.M.M.T.B. Herath

**Affiliations:** a Kandy General Hospital, Sri Lanka; b National Hospital of Colombo, Sri Lanka

**Keywords:** Evidence-based medicine, Infectious disease, Internal medicine

## Abstract

Corticosteroids are used therapeutically for a broad spectrum of diseases including autoimmune, allergic and inflammatory diseases. However in trials, the evidence for using corticosteroids in dengue is inconclusive and the quality of evidence is low. This systemic review is conducted to review clinical trials on dengue and steroid therapy to identify the current strength and weakness of evidence for the use of corticosteroids.

We searched MEDLINE/PUBMED and Google scholar for publications on steroid use in dengue and the relevant authors of the study were contacted for additional information, as required. This review includes thirteen studies enrolling 1293 children and adult participants. There was no evidence of viremia and no significant side effects after the administration of low and high doses of oral corticosteroids and high doses of intravenous corticosteroids. Beneficial therapeutic effects were seen in some studies, which used high doses or multiple doses of steroids.

The effectiveness of corticosteroids in dengue is depended upon sustained therapeutic blood levels of corticosteroids for an adequate duration and using a steroid with higher receptor affinity. Further clinical trials using pharmacologically and immunologically accepted standard steroid protocols are warranted to validate this conclusion.

## 1 Introduction

World Health Organization (WHO) has published guidelines for the management of dengue fever. This management prevents hemoconcentration during the early phase and fluid overload in the late phase of the illness and halts severe complications such as dengue shock syndrome (DSS) and multiple organ failure. Improved fluid management protocols have resulted in a large decrease in mortality in dengue infection (Wongsa, 2015). However this approach does not consider any immune suppressive therapy to prevent immunological damage in dengue hemorrhagic fever (DHF), DSS and other complications in dengue such as carditis, ascites, liver and renal damage, encephalitis and bleeding.

Corticosteroids are potent immune modulators and are used therapeutically for a broad spectrum of diseases including autoimmune, allergic and inflammatory diseases (Berkovich, 2013; Ruiz-Irastorza et al., 2012; Sinha and Bagga, 2008). However, the evidence from trials using corticosteroids in dengue is inconclusive and the quality of evidence is low to very low. Cochrane Database Systemic Review 2014 concluded that evidence is insufficient to evaluate the effects of corticosteroids in the treatment of early stage dengue fever and dengue-related shock (Zhang and Kramer, 2014). However when immune-mediated mechanisms, cross-reacting antibodies, cytokines and chemokines (Leong et al., 2007; Watanabe et al., 2015) are considered, the immune pathology of dengue has many similarities to other autoimmune diseases which have been treated effectively by corticosteroids for several decades. Clinical trials have shown supportive evidence for the actions of corticosteroids in dengue fever (Futrakul et al., 1987; Futrakul et al., 1981; Min et al., 1975; Premaratna et al., 2011; Tam et al., 2012; Villar et al., 2009). Interestingly there was no evidence of viremia in three clinical trials (Nguyen et al., 2013; Tam et al., 2012; Villar et al., 2009) and no significant side effects after the administration of low and high oral doses of oral corticosteroids and high doses of intravenous (IV) corticosteroids in two trials (Tam et al., 2012; Villar et al., 2009). Therefore a reasonable argument exists regarding corticosteroid use in dengue and this should be investigated. Therefore this systemic review is conducted on the past researches done on dengue and steroid therapy to identify the strength and weakness of evidence for the use of corticosteroids.

## 2 Main text

This study is aimed to compare the treatment protocol of steroids in dengue clinical studies with a known or gold standard protocols used in other autoimmune disorders and to identify the factors influencing the use of steroids in dengue immune pathology. We searched MEDLINE/PUBMED and Google scholar for publications with the search terms ‘dengue’ and ‘steroid’, ‘corticosteroid’, ‘prednisolone’, ‘methylprednisolone’ or ‘dexamethasone’ in the title and abstract, up until 4 January 2018. Authors independently screened the identified titles and abstracts and retrieved full-text articles of potentially relevant trials. The identified full-text articles were screened for the eligibility to decide on the final list of studies. Some of the authors were contacted for clarification, if the eligibility was unclear. Then authors independently extracted the available data using a pre-specified, pre-piloted data extraction form. Two authors independently extracted and crosschecked the data to minimize errors. The number of trials with standard steroid protocol was insufficient to construct a funnel plot to assess publication bias.

The participants with dengue were analyzed according to the stage (preliminary stage – from the onset of the dengue symptoms to the earliest plasma leakage stage, intermediate stage – from the onset of the critical phase to the time before the severe stage of DSS, late stage – severe profound shock) and the steroid type {methylprednisolone (MP), hydrocortisone (H), dexamethasone (DX) and prednisolone (P)}, route of administration and doses. In the analysis, primary outcome was death and the secondary outcomes were measures of severity (need for blood transfusion, complications, haemorrhages, duration of shock, duration of hospitalization, bleeding, severe thrombocytopaenia, ascites, intensive care unit (ICU) admission, viremia, haematocrit, white blood cell count, platelet count, host immune markers).

Three grading were used in this review; grading of steroid protocol (GS), grading of recommendations (GR) (Ansari et al., 2009; “Making use of guidelines in clinical practice; implementing clinical guidelines: A practical guide,” 1999) and grading of evidence (GE).

### 2.1 Grading of steroid protocol (GS)

There are no consensus regarding the minimal effective dosage, length of treatment and the route of administration for systemic corticosteroid treatment in DF, DSS and DHF, though it was graded from A to E in this review. The premier quality is considered as grade A. Thereafter quality is tapered gradually until reaching grade E. In practice, a variety of treatment regimens of steroids for DSS and DHF have been adopted based largely on the clinical experience, investigation findings and many clinical trials in dengue performed over the last 50 years. In addition, standard steroid régime used in autoimmune and allergic disorders such as Systemic lupus erythematosus (SLE), multiple sclerosis, acute asthma (Berkovich, 2013; Ruiz-Irastorza et al., 2012; Sinha and Bagga, 2008), findings and experience from other clinical trials were also considered here for grading of steroid protocol.

A. High dose IV methyl prednisolone daily for three to five days.
B. One high dose IV methyl prednisolone
C-1. High dose oral prednisolone for at least 3 days.
C-2. Low dose oral prednisolone at least 3 days.
D-1. One high dose of IV Hydrocortisone
D-2. IV Hydrocortisone 50 mg four hourly three days.
D-3. IV hydrocortisone for 3 days in tapering manner
D-4. Several dose of IV Hydrocortisone 30 mg
E. IV 4 mg dexamethasone, followed by 2 mg doses every 8 hourly for 24 h or IV dexamethasone 8 mg initially, followed by 4 mg every 8 hourly for 4 days

### 2.2 Grading of evidence (GE)

I. Evidence is based on randomized controlled trials (or meta-analysis of such trials) of adequate size to ensure a low risk of incorporating false-positive (alpha) or false-negative (beta) results.

II. Evidence is based on randomized controlled trials that are too small to provide ‘level I’ evidence. They may show either positive trends that are not statistically significant or no trends and are associated with a high risk of false-negative results.

III. Evidence is based on non-randomized controlled or cohort studies, case series, case-control studies or cross-sectional studies. III-1. A pseudo randomized controlled trial I, III-2. A comparative study with concurrent controls, III-3. A comparative study without concurrent controls.

IV. Evidence is based on non-randomized, historical controls and expert opinion, case series with either post-test or pre-test/post-test outcomes.

### 2.3 Grading of recommendations (GR)

A- Supported by at least two, level I evidence.
B- Supported by one, level I evidence.
C- Supported by level II evidence only.
D- Supported by at least one, level III evidence.
E- Supported by level IV evidence.

This review includes thirteen studies enrolling 1293 children and adult participants. For explanatory and analytical purposes the total period of dengue illness is divided into three phases: preliminary stage, early stage and late stage. The clinical trials done so far have been categorized into these three stages and summarized in Tables 1, 2, and 3. In this summary tables, grading are categorized under first column. The corresponding authors, year of the study done, age range and the number of participants are in the second column and the steroid doses and the duration of the drug are in the third column. The fourth column includes the reason for favoring or not favoring for steroids, other findings and conclusions. Explanations and other findings are shown into last column. Out of these studies, five were done in preliminary stage that enrolled 831 participants, five studies were done in the intermediate stage with 316 participant and three studies were done in the late stage that enrolled 146 participants. The trials have been conducted in Vietnam, Columbia, India and Sri Lanka.

1. The use of corticosteroids at the preliminary stage (Table 1)

**Table 1:** The use of corticosteroids at the preliminary stage.

Grading of evidence (GE), steroid protocol (GS), Recommendation (GR)	Authors/Reference/Study year/number/age of participants/Shock stage of recruited group	Dose and duration of steroid	Results/conclusion	Explanation for the results
GE-II GS-C 1, C2 GR-C	Tam DT et al./(Tam et al., 2012)/2012/255/Adult and children/No Shock	Low-dose (0.5 mg/kg) or high-dose (2 mg/kg) oral prednisolone therapy for 3 days. Within dengue fever for ≤72 hours.	No/no significant adverse effects or prolongation of viremia were seen. No reduction in the incidence of shock.	High-dose could reduce the risk of shock by up to 43%.
GE-II GS-B GR-C	Villar LA et al./(Villar et al., 2009)/2009/189/Adult and children (age group 5–15 years and >15/No Shock)	IV MP 15 mg/kg single dose, within 120 hours of onset of fever	Yes/reduces the incidence of bleeding and no prolongation of viremia and no significant adverse events	MP has the highest receptor affinity out of corticosteroids and IV root access is used. Treated early in the cause of illness.
GE-III GS-E GR-D	Kularatne S.A.M. et al./(Kularatne et al., 2009)/2009/100/adult/No Shock	IV 4 mg dexamethasone, followed by 2 mg doses every 8 hourly for 24 hours	No/A low dose is used, dexamethasone was not effective in achieving a higher rise of platelet count in dengue infection	Four-day course of high-dose dexamethasone (40 mg/day) is an effective dose. Dexamethasone 10 mg/day was used in this study
GE-III-1 GS-E GR-D	Shashidhara K.C. et al./(Shashidhara et al., 2013)/2013/62/adults/No Shock	IV dexamethasone 8 mg initially, followed by 4 mg every 8 hourly thereafter for 4 days	No/A low dose x used, dexamethasone was not effective in achieving a higher rise of platelet count in dengue infection	Four-day course of high-dose dexamethasone (40 mg/day) is an effective dose. Dexamethasone 12 mg/day was used in this study
GE-III-1 GS-C1, C2 GR-D	Thi Hanh Tien Nguyen et al./(Nguyen et al., 2013)/2013/225/children and young adults (aged 5–20 years)/No Shock	Low-dose (0.5 mg/kg) or high-dose (2 mg/kg) regimens of oral prednisolone/Fever for less than 72 hours	No/Early prednisolone therapy has little impact on the host immune response or the clinical evolution of dengue	After corticosteroid administration, It may take longer duration to detect changes of immune markers. In this study, it was checked only day 1 and day 2

**Table 2:** The use of corticosteroids during intermediate stage.

Grading of evidence (GE), steroid protocol (GS), Recommendation (GR)	Authors/Reference/Study year/number/age of participants/Shock stage of recruited group	Dose and duration of steroid	Results/conclusion	Explanation for the results
GE-III-2 GS-D-2 GR-D	Fernando S et al./(Fernando and Samarawickrama, 2016)/2015/100/Adult/Dengue hemorrhagic fever at grade I and grade II	IV HC 50 mg 4 times per day for three days	Yes/92 % had improved within 72 hours/24% of control found to have myocarditis, hemorrhage, pneumonia	Pharmacologically effective drug protocol was used in the trial that maintained therapeutic drug levels.
GE-II and III -2 GS-D3 GR-D	Min M et al./(Min et al., 1975)/1975/98/Children./shock	IV HC as follows: day 1: 25 mg/kg, day 2:15/kg, day 3:10 mg/kg, for 3 days	Yes/a statistically significant mortality benefit with steroid	Pharmacologically effective drug protocol was used in the trial.
GE- III GS-D1 GR-D	Dummy Sumarmo, et al./(Sumarmo et al., 1982)/1982/87/Children 8 years/shock	A single dose of IV HC hemisuccinate, 50 mg/kg.	No/No value in the treatment hydrocortisone	No sustained effective drug dose was maintained. High dose effect last only for a short period. HC has low receptor affinity than MP.
GE-III-2 GS-A GR-D	Futrakul P et al./(Futrakul et al., 1981)/1981/22/children 6 months to 14 years	IV MP: 10–30 mg/kg day. Single or repeated dose	Yes/9 out of 11 treatment group survived. All patients in the control groups died	Sustained and effective drug dose was maintained. Single dose may help due to higher receptor affinity of MP
GE-III-2 GS-A GR-D	Futrakul P et al./(Futrakul et al., 1987)/1987/9/Children 2.5–13 years	IV MP: 30 mg/kg day. Repeated dose were given to 7 patients	Yes/Significant hemodynamic improvement.	Sustained and effective drug dose was maintained with using a drug with higher receptor affinity.

**Table 3:** The use of corticosteroids during the late stage.

Grading of evidence (GE), steroid protocol (GS), Recommendation (GR)	Authors/Reference/Study year/number/age of participants/Shock stage of recruited group	Dose and duration of steroid	Results/conclusion	Explanation for the results
GE-III-1 GS-B GR-D	Premaratna R. et al./(Premaratna et al., 2011)/2011/55/Adult/Sever shock stage	IV MP 1 g signal dose.	Yes/Hematological recovery, hospital stay, morbidity after recovery were all significantly shorter in the corticosteroid groups.	Single high dose of a drug with higher receptor affinity and low mineralocorticoid action is used. Reduced confounding factors due to better fluid management in 2011 than 1993 and 1875.
GE-II GS-B GR-C	Tassniyom et al./(Tassniyom et al., 1993)/1993/63/Children, 15 years/profound shock	IV MP signal dose 30 mg/kg.	No./Did not reduce mortality in severe dengue shock syndrome, pneumonia, convulsion, cardiac arrest, pulmonary hemorrhage and positive hemoculture	At profound shock stage of the illness body does not respond to conventional critical care. Fluid management methods might not be better established in 1993 than 2011 that might have masked the benefit effect of Corticosteroids.
GE-III-3 GS-D4 GR-D	Widya MS. et al./(Widya and Martoatmodjo, 1975)/1975/28/children/Most patients profound shock	IV HC 30 mg 4–6 hourly (120–180 mg per day).	No/No effects of corticosteroids in severe dengue shock syndrome.	Hypervolemia due to mineralocorticoid action of HC could cause increase mortality and morbidity at this stage.

This period is from the onset of the dengue symptoms to the earliest plasma leakage stage. The study conducted by Tam D.T. et al., provides level II evidence (Tam et al., 2012). 255 Vietnamese patients, aged between 5 to 20 years, with dengue fever for less than 72 hours and prior to the onset of the critical phase participated in this trial. Two selected groups in the study were treated with low-dose (0.5 mg/kg) and high-dose (2 mg/kg) oral prednisolone therapy separately for 3 days. The study's final conclusion was that use of oral prednisolone during the early stage of dengue infection was neither associated with significant adverse clinical or virological effects nor with a reduction in the incidence of recognized complications of dengue. In contrast it was highlighted that a high-dose of steroids could reduce the risk of shock by up to 43%.

Villar L.A. et al., and colleagues also conducted a level II study on patients in the age group of 5–15 years and more than 15 years (Villar et al., 2009) with administration of a IV single dose of 15 mg/kg methyl prednisolone. This study found that there was a reduction in the incidence of bleeding and ascites with steroids. A Level –II study was conducted by Thi Hanh Tien Nguyen et al., by administering high-dose prednisolone (2 mg/kg) and low-dose prednisolone (0.5 mg/kg) for 3 days (Nguyen et al., 2013). In his study, the concentrations of 11 cytokines and chemokines were measured on each day for three days. It was found that acute-phase plasma cytokine were not significantly attenuated by prednisolone treatment during the acute phase of illness. They were also unable to demonstrate any reduction in the severity of plasma leakage or immune-modulation with prednisolone. However prolonged viremia was not observed in prednisolone treated patients in this study as well. Two clinical trials to assess the rise of platelet count following low and high doses of dexamethasone concluded that low (6 mg/day) or high doses of dexamethasone (12 mg/day) was ineffective in increasing the platelet count in dengue (Kularatne et al., 2009; Shashidhara et al., 2013).

Summarizing the studies in preliminary stage, two trails using high and low doses of prednisolone for 3 days (GR –C, GE-II and GR- D, GE-III-1) and two trails using IV dexamethasone (GR-D, GE-III and GR-D, GE-III-1) did not show beneficial effect of steroids. Only one study which used single dose of IV methylprednisolone (GR-C, GE-II) showed a beneficial effect of steroid. In this stage, there was no evidence of viremia (two trials; 444 participants with level II evidence and one trial; 225 participants with level III-1 evidence) and no significant side effects after the administration of low and high oral doses of corticosteroids and high doses of IV corticosteroids (two trials; 444 participants with level II evidence).

2. The use of corticosteroids during the intermediate stage (Table 2)

In this review, the intermediate stage is considered from the onset of the critical phase to the time before the severe stage of DSS. This does not include grade IV stage of DHF or profound DSS.

In one study, conducted by Fernando S. and colleagues (Level III-2 evidence) used hydrocortisone in dengue patients in the category of DHF grade I and grade II (Fernando and Samarawickrama, 2016). It showed early recovery after administration of 50 mg IV hydrocortisone, 4 times per day, for three days. Among them, 92% had improved within 72 hours. All improved without going into advanced leaking phase. 50 participants in the control group were treated strictly adhering to standard dengue management protocols. 24% of them developed complications such as myocarditis, hemorrhage and pneumonia. Another study conducted by Min and his colleagues (Level II and III-2 evidence) showed the beneficial effects of corticosteroids (Min et al., 1975). They used a tapering dose of IV hydrocortisone as follows: day 1–25 mg/kg, day 2–15/kg and day 3–10 mg/kg, for 3 days for older children (8 years and over). A statistically significant mortality benefit with steroids was observed in this trial. Moreover, two trials with Level III-3 evidence conducted by Futrakul P. and colleagues in 1981 and in 1987 found beneficial effects after administration of one or more repeated doses of IV methylprednisolone 10–30 mg/kg (Futrakul et al., 1981, 1987).

One clinical study without administration of repeated doses of steroids, showed no beneficial effect of corticosteroids in this stage. In this study, a single dose of hydrocortisone (50 mg/kg of body weight (equal to 10 mg/kg MP) was administrated to children with dengue shock syndrome). The response to therapy was virtually identical in 47 children who were treated with steroids to 50 children who were not. In this trial mortality, duration of shock and the fluid requirement were also measured. It was concluded that hydrocortisone is of no value in the treatment of dengue shock syndrome (Sumarmo et al., 1982).

All five trials in intermediate stage were in the GR –D and were close to or at GE-III levels. Four trials, which used intravenous hydrocortisone or methylprednisolone multiple doses, showed marked beneficial effect of steroid. However one trial, which used single dose of intravenous hydrocortisone, showed no beneficial effect of steroid.

3. The use of corticosteroids during the late stage (Table 3)

Beneficial effects of Corticosteroids was shown by Premaratna R. et al., by administering a single dose of IV methylprednisolone 1 g (equal to 5000 mg of hydrocortisone) to adult patients in severe DSS stage (Premaratna et al., 2011). They found that hematological recovery; hospital stay and morbidity after recovery were all significantly shorter in corticosteroid treated group. Conversely, Tassniyom and his colleagues concluded that a single high dose of methylprednisolone did not reduce mortality in severe dengue shock syndrome (Tassniyom et al., 1993). In this trial a single dose of IV methylprednisolone (30 mg/kg) were given to sixty-three children with profound dengue shock. They also found that complications such as occurrence of fever after shock, pneumonia, convulsion, cardiac arrest, pulmonary hemorrhage and positive hemoculture were not significantly different in the treatment and control groups. Another study was done by Widya M.S. and Martoatmodjo in 1975, in which children with severe dengue shock were administered 30 mg IV hydrocortisone, 4–6 hourly/day (Widya and Martoatmodjo, 1975). Ten patients died out of a total of 28 patients. Thus they concluded corticosteroid is ineffective in severe dengue.

In summary two trials in late stage, which used single intravenous methylprednisolone dose (GR-C and GE-II) and multiple low doses intravenous hydrocortisone (GR-D and GE-III-3) did not show beneficial effects. However another study using intravenous single dose of methylprednisolone (GR-D and GE-III-1) showed beneficial effects.

When considering these studies, most of the beneficial results were seen with intravenous usage, with methyl prednisolone, with high doses and with multiple doses. At high concentrations glucocorticoid molecules intercalate into cell membrane and alter cellular functions resulting in reduced calcium and sodium cycling across the plasma membranes of immune cells. This is thought to contribute to rapid immunosuppression and a subsequent reduction of the inflammatory process when corticosteroids are used in high concentration (Sinha and Bagga, 2008). Furthermore, considering the pharmacological aspect, the low-dose steroids may act via the genomic pathway of corticosteroids, whereas a high-dose of steroids may act through both genomic and non-genomic pathways (Sinha and Bagga, 2008). When only a single glucocorticoid application is used, the effect would last only for a short duration because receptor occupation rapidly reverts to the original value. This is the importance of multiple doses to have a sustained effect (Schimmer and Parker, 2006). Methylprednisolone may have a quicker penetration of the cell membrane and intravenous methylprednisolone shows a rapid peak (Ito et al., 1992).

If asthma is taken for comparison it is recommended to use medium or high dose of systemic corticosteroids for about 5 days in acute asthma exacerbations to reduce the rate of relapse and for the improvement in lung function (Alangari, 2014). In the above mentioned studies it should be noted that the steroids had not been continued for more than 3 days. This might be a reason for not having a beneficial effect, especially in trials in which steroids is used during the early stage of dengue fever, since the severe dengue immune pathology starts after three to five days from the onset. Furthermore, immune involvement in dengue extends to multiple-organs and more inflammatory markers appear in the body compared to asthma, which is mainly limited to the lungs. Thus the doses of corticosteroids in dengue management should be higher than the dose for acute asthma and the duration of administration must also be longer than average. Even in immune thrombocytopenia (ITP), which has an immune mediated pathology causing low platelets, high-dose intravenous methylprednisolone have more beneficial effects (Alpdogan et al., 1998; Ozsoylu et al., 1989; Visser et al., 1999). The reason for not having a beneficial effect in two trails, which used dexamethasone, again can be due to inadequate doses for shorter duration. Even in ITP, it is preferred to use high dose dexamethasone for an adequate time (dexamethasone 40 mg/d for four days) as a first line treatment (Wei et al., 2016), which is higher than the doses used in these studies.

Another important observation is that the most beneficial effects of the steroids were seen in the intermediate stage. This may be because the immune-mediated mechanisms, cross-reacting antibodies, cytokines and chemokines are high during this period, which can be suppressed by adequate steroid use. In the late stage even though one study (Premaratna R, 2011) (Premaratna et al., 2011) has showed beneficial effects of intravenous methylprednisolone, other study (Tassniyom, 1993) (Tassniyom et al., 1993) did not show this same results. One possibility is, since profound DSS is the most severe stage of the disease, there would not have been enough time as well as suitable body condition to activate biological actions of steroids properly and adequately. Severe tissue damage, hypotension, hypoxia, prolonged shock, acidemia, electrolyte and PH imbalance and subclinical infections could mask the effects of corticosteroids. In addition, new fluid management guidelines may not have been well established in 1993 (during the time of Tassniyom) as in 2011 (during the time of Premaratna R) to avoid confounding factors against beneficial effects of steroid. These factors could have affected the results of the study of Tassniyom and masked the benefits of corticosteroids. However it can be beneficial to have a standard drug protocol for this life threatening late stage of dengue similar to what is used in the critical stages of autoimmune diseases. For instance, the gold standard drug protocol used in organ and/or life-threatening manifestations of SLE is 1 g/day of methylprednisolone for 3 consecutive days (Badsha and Edwards, 2003). Using intravenous hydrocortisone during this stage has shown no benefit (Widya and Martoatmodjo, 1975). This may be because the mineralocorticoid effect of several doses of hydrocortisone might have led to hypervolemia and detrimental effects.

## 3 Conclusion

It is highly unlikely that use of corticosteroids in dengue treatment results in viremia, any other significant adverse effects or complications. In fact, the effectiveness of corticosteroid in dengue is depended upon sustained and maintained therapeutic blood levels of corticosteroids for an adequate duration and using a steroid with higher receptor affinity. Further clinical trials using pharmacologically and immunologically accepted standard steroid protocols are warranted to validate this conclusion. It is also important to note that individual patients may respond differently so it is difficult to make general guidelines to all patients.

## Declarations

### Author contribution statement

All authors listed have significantly contributed to the development and the writing of this article.

### Funding statement

This research did not receive any specific grant from funding agencies in the public, commercial, or not-for-profit sectors.

### Competing interest statement

The authors declare no conflict of interest.

### Additional information

No additional information is available for this paper.
